# Microplastic burden in invasive signal crayfish (*Pacifastacus leniusculus*) increases along a stream urbanization gradient

**DOI:** 10.1002/ece3.10041

**Published:** 2023-05-03

**Authors:** Abigail R. Dent, Daniel D. A. Chadwick, Lawrence J. B. Eagle, Alex N. Gould, Matthew Harwood, Carl D. Sayer, Neil L. Rose

**Affiliations:** ^1^ Department of Geography University College London London UK; ^2^ PBA Applied Ecology Settle, North Yorkshire UK

**Keywords:** freshwater ecosystem, macroinvertebrate, microplastics, *Pacifastacus leniusculus*, signal crayfish, urbanization

## Abstract

Microplastics are a globally pervasive pollutant with the potential to directly impact species and accumulate in ecosystems. However, there remains a relative paucity of research addressing their accumulation in freshwater ecosystems and a near absence of work in crayfish, despite their high ecological and economic importance. This study investigated the presence of microplastics in the invasive signal crayfish *Pacifastacus leniusculus* along a stream urbanization gradient. The results demonstrate a ubiquitous presence of microplastics in crayfish digestive tracts at all sites and provide the first evidence of microplastic accumulation in tail tissue. Evidence of a positive linear trend was demonstrated between microplastic concentration in crayfish and upstream urban area size in generalized linear models. Evidence for a positive effect of the upstream urban area and a negative effect of crayfish length on microplastic concentrations in crayfish was demonstrated in multiple generalized linear regression models. Our results extend the current understanding of microplastics presence in freshwater ecosystems and demonstrate their presence in crayfish in the wild for the first time.

## Graphical abstract

This study investigated the presence of microplastics in the invasive signal crayfish *Pacifastacus leniusculus* along a stream urbanization gradient. The results demonstrate a ubiquitous presence of microplastics in crayfish digestive tracts at all sites and provide the first evidence of microplastic accumulation in tail tissue. Our results extend the current understanding of microplastics presence in freshwater ecosystems and demonstrate their presence in crayfish in the wild for the first time.

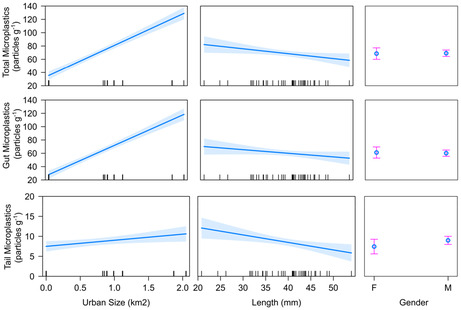

## 1 INTRODUCTION

Global plastic production and usage continue to grow (Lebreton & Andrady, 2019), with rates of production exceeding 330 million tons per year (Jiang et al., 2019; Talbot & Chang, 2022). Plastic is a low‐cost, versatile, and extremely durable material making it a useful societal resource (Chamas et al., 2020; Walkinshaw et al., 2020). However, some of its properties, including its durability and resistance to degradation, are of major environmental concern (Chamas et al., 2020; Cole et al., 2011; Geyer et al., 2017).

Some of the most pervasive and concerning forms of plastic in the aquatic environment are microplastics. Microplastics, often defined as plastics <5 mm in size (Horton et al., 2017), were identified in marine environments as early as the 1970s (Carpenter & Smith, 1972) and currently have a near‐global contemporary distribution (Eerkes‐Medrano et al., 2015; Rummel et al., 2017; Wagner et al., 2014; Woodall et al., 2014).

Much of the research on understanding the distribution, density, and chronic impact of microplastics have focused on the marine environment, despite reported evidence that freshwater environments have comparable microplastic concentrations (Eerkes‐Medrano et al., 2015). Further, current freshwater research is generally focused on microplastic presence in fishes (Biginagwa et al., 2016; Sanchez et al., 2014) and birds (D'Souza et al., 2020; Gil‐Delgado et al., 2016; Reynolds & Ryan, 2018). Fewer studies have investigated microplastic concentration in lower trophic organisms, such as freshwater crayfish (Chen et al., 2020; Lv et al., 2019; Zhang, Fraser, et al., 2021), despite their high ecological and economic importance (Harlıoğlu & Farhadi, 2017; Reynolds et al., 2013). Many crayfish species exhibit polytrophic, omnivorous feeding behaviors (Chucholl, 2013; Jackson et al., 2014), acting as keystone species (Holdich et al., 2009, 2014). As such, crayfish are well positioned to act as an important conduit of microplastic pollution throughout freshwater ecosystems (Alford et al., 2017; Jiang & Cao, 2021).

There have been some efforts to investigate microplastic presence in crayfish in China (Chen et al., 2020; Lv et al., 2019; Zhang, Fraser, et al., 2021). Lv et al. (2019) and Zhang, Fraser, et al. (2021) detected microplastics in water, sediment, and Red Swamp crayfish *Procambarus clarkii* from isolated rice paddies and controlled freshwater aquaculture ecosystems, respectively. Similar microplastic loads were recorded in water and sediment samples, and in gill, stomach, and gut samples from study pond and rice‐crayfish co‐culture systems (Zhang, Fraser, et al., 2021). However, these studies did not report microplastics in flesh samples. Recent work on Redclaw crayfish *Cherax quadricarinatus* indicates the consumption of such microplastics can have ecotoxicological effects altering crayfish gene expression, enzyme production, and thus metabolic processes (Chen et al., 2020). Consequently, there is a need to identify whether microplastic ingestion is a common occurrence across other globally abundant crayfish species.

While research on microplastics in freshwaters has recently received increasing attention (Bigalke et al., 2022; Liu et al., 2022; Wu et al., 2022; Xiang et al., 2022), there remains a notable absence of studies addressing microplastics in crayfish and western fluvial systems. We investigated the presence of microplastics in invasive signal crayfish *Pacifastacus leniusculus* populations in streams situated in North Yorkshire, Northern England, UK. The signal crayfish was introduced into the UK in the 1970s for aquaculture (Holdich & Rogers, 1997), with a present‐day distribution across the majority of England (Chadwick, 2019; Holdich & Reeve, 1991) and wide‐ranging impacts on aquatic ecosystems (Vaeßen & Hollert, 2015). However, the distribution of microplastics in crayfish in the UK has so far received little attention despite the potential for transfer through freshwater trophic pathways. Thus, driven by the knowledge that plastic pollution is linked to the size of an urban area and catchment population density (Lebreton et al., 2017; Strokal et al., 2021), we compared microplastic pollution within water and signal crayfish along stream urbanization gradients.

## 2 MATERIALS AND METHODS

### 2.1 Study area

**FIGURE 1:**
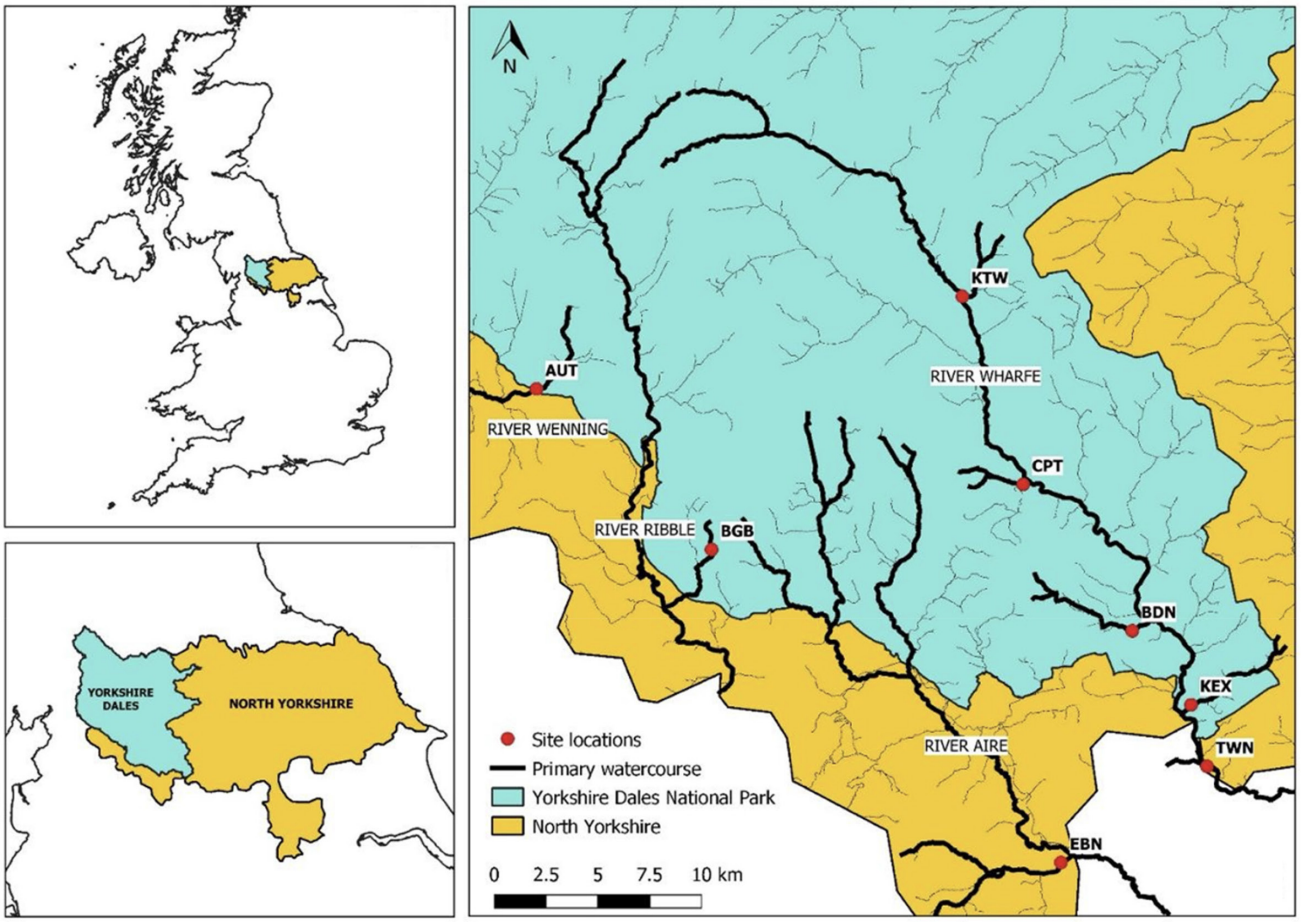
Location of sampled watercourses within the Yorkshire Dales National Park, North Yorkshire, Northern England. Single column fitting image.

The study was conducted in North Yorkshire, England. Study sites were located across the River Wharfe, Ribble, Aire, and Wenning catchments within the Yorkshire Dales National Park and surrounding environment (Figure 1). Eight study sites were selected downstream of urban conurbations of varying sizes to establish an increasing gradient of both upstream urban area (maps) and size of the human population (UK Gov, 2011) within each catchment (Table 1). The urbanization gradient was defined by the extent of the urban area (km^2^) and the size of the human population upstream of a site. A control site (Bookill Gill Beck) (Table 1) was included, which has a catchment dominated by unimproved and semi‐improved pastures with almost no upstream semi‐urban land, enabling calculation of baseline microplastic concentrations for the region. Each site was known to have a well‐established population of invasive *P. leniusculus*, and no modern records of native white‐clawed crayfish *Austropotamobius pallipes*. No native crayfish were encountered.

**TABLE 1:** Key descriptors of the eight sampled watercourses flowing from the Yorkshire Dales National Parks (italics indicate control site).

Catchment	Beck name	Code	Size of urban area (km^2^)	Human population of upstream urban area (individuals)
Ribble	*Bookill Gill*	BGB	0.00	0
Wharfe	Barden	BDN	0.83	107
Wharfe	Kex	KEX	0.85	132
Wharfe	Kettlewell	KTW	0.89	334
Wenning	Austwick	AUT	0.99	553
Wharfe	Captain	CPT	1.12	649
Wharfe	Town	TWN	1.87	3644
Aire	Eastburn	EBN	2.05	7967

### 2.2 Sample collection and preparation

The study sites were sampled between 19 and 28 May 2021, comprising a single night of trapping, followed by water sampling and supplementary handsearching where required. Sampling followed Check‐Clean‐Dry best practice guidance with all equipment disinfected (FAM 30 Iodophor). Trapping was authorized by the Environment Agency (CR1 license) and undertaken with landowner permissions. Sites were selected on the criteria of being within 1 km downstream of the identified urban areas within each catchment and with safe riparian access.

At each site triplicate 0.5 L water samples were filtered on‐site through glass microfiber filters (Whatman™, 1.2 μm particle retention). The metal‐lined filtration system was rinsed with site water three times presampling and capped underwater to avoid atmospheric contamination.

At each study site, *P. leniusculus* were collected via fladen crayfish traps (500 mm × 200 mm; entrance diameter: 50 mm; mesh size: 5 mm) and handsearching. All *P. leniusculus* caught (*n* = 41) were transferred to sterilized cool boxes and then frozen. Once humanely euthanized, *P. leniusculus* samples were thawed and washed with deionized water to remove microplastic contamination from the exoskeleton. Foreguts, hindguts, and tail muscle tissues were dissected out of each specimen (*n* = 123) and were freeze‐dried at −50*°*C for 72 h (Edwards). The dry mass of each sample was subsequently recorded (±0.01 g). Hydrogen peroxide (30%) was then added to each crayfish tissue sample during heated centrifuging (30 min at 75*°*C) until no organic material remained (adapted from Masura et al., 2015). Reagent‐grade sodium chloride and deionized water were subsequently added until all sodium chloride had dissolved to neutralize the solution. After digestion, samples were individually filtered through the same glass microfiber filter papers as used for the water samples using a Multiple Vacuum Filtration System (Membrane Solutions) and then dried in a drying cupboard (24 h at 30°C). Procedural blanks were run for crayfish (*n* = 7) and water (*n* = 3), in addition to a positive microplastic control (*n* = 1); procedural blanks indicated negligible contamination (*p* < .001). All sample processing was undertaken in a horizontal laminar flow cabinet to avoid exogenous contamination, and clothing made from man‐made materials was limited to prevent contamination. At each stage of the research, equipment and workbenches were cleaned thoroughly to prevent microplastic cross‐contamination.

All filter paper contents were examined at 40× magnification using a LEICA S6 D Stereo Zoom Microscope attached to a ZEISS Axiocam ERc 5 s camera (ZEISS). Microplastics were visually grouped by type (“fiber,” thread‐like polymers; “fragment,” jagged‐edged pieces of larger materials; or “film,” a flat, thin, often transparent sheet) and classified into a color category. Microplastic fibers were identified against Rochman et al. (2019) reference images and then enumerated. To confirm microplastic counts were consistent, a subsample (>10%) of randomly selected filter papers (*n* = 15) was re‐examined for microplastic. No evidence for a difference was found between original and re‐examined filter papers (*t*(14) = 6.42, *p* = .531). Concentrations of microplastic were calculated as particles per gram of dry weight for *P. leniusculus* samples and particles per 100 mL for water samples.

Suspected microplastic fibers and fragments from a randomly selected subsample of *P. leniusculus* filter papers (*n* = 24) and water sample filter papers (*n* = 16) were analyzed using micro‐Fourier Transform Infrared (μFT‐IR) Spectroscopy. This approach follows the current practice in the literature (Lv et al., 2019; Tien et al., 2020; Wardlaw et al., 2022). Colored fibers and fragments (*n* = 79) were individually analyzed under a Nicolet™ iN10 MX Infrared Imaging Microscope (Thermo Scientific) to determine the polymeric composition; match similarity scores ≥70% were deemed reliable. Analyses were performed in reflectance mode with a cooled detector. Spectra were collected from an average of 16 sample scans in the wavelength range 675–4000 cm^−1^ at a resolution of 4 cm^−1^. Background spectra were generated before the sample. μFT‐IR spectra obtained were compared with a polymer library, compiled at University College London, in the OMNIC Picta Software. The validation procedure included procedural positives, in which known plastic pieces were processed in the same manner as suspected plastics from samples.

### 2.3 Statistical analyses

The two measures of urbanization, total population size (individuals) and urban area (km^2^), were recorded and were highly correlated (Pearson's rho 0.833, *p* = .010), and as such, only urban area was included in subsequent analyses. Generalized linear models with Gaussian error distributions were used to analyze relationships between microplastic concentrations in water and *P. leniusculus* samples against urban area. The relationship between mean water sample microplastic concentration and urban area was explored. The relationship between mean total, gut (foregut and hindgut), and tail microplastic concentrations and urban area (km^2^) were explored. Tail microplastic samples from Town Beck crayfish were omitted from analyses due to the unsuitability of the processed samples.

Multiple generalized linear regression with Gaussian error distributions were used to relate total, gut, and tail microplastic concentrations in individual crayfish to predictor variables upstream urban area, carapace length, and gender. In this study, no interaction effects were assumed when undertaking multiple regression.

All statistical analyses were performed using SPSS (v 27.0) and R (v 3.5.1; R Core Team, 2018). All graphs and tables were generated in R and Excel (v 16.52). Scatter plots were produced in base R (R Core Team, 2018) and effect plots for multiple regression were produced using the effects package (Fox & Weisberg, 2018). For all statistical analyses, an evidence‐based language was adopted for reporting the results (Muff et al., 2022) alongside traditional significance reporting.

## 3 RESULTS

### 3.1 Microplastic occurrence, identification, and composition

**FIGURE 2:**
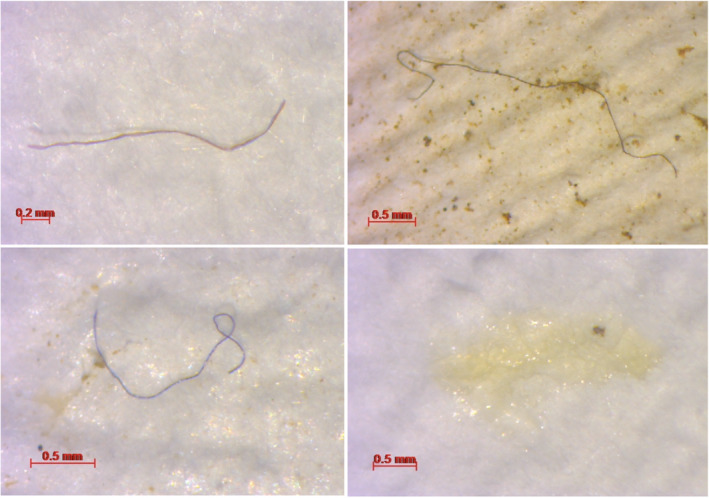
Photographs of suspected microplastics using the ZEISS Axiocam ERc 5 s camera, objective 40×: (a) red fiber from a Captain Beck *P. leniusculus* sample, (b) black fiber from a Kettlewell Beck water sample, (c) blue fiber from an Eastburn Beck *P. leniusculus* sample and (d) transparent film from a Kex Beck *P. leniusculus* sample.

**FIGURE 3:**
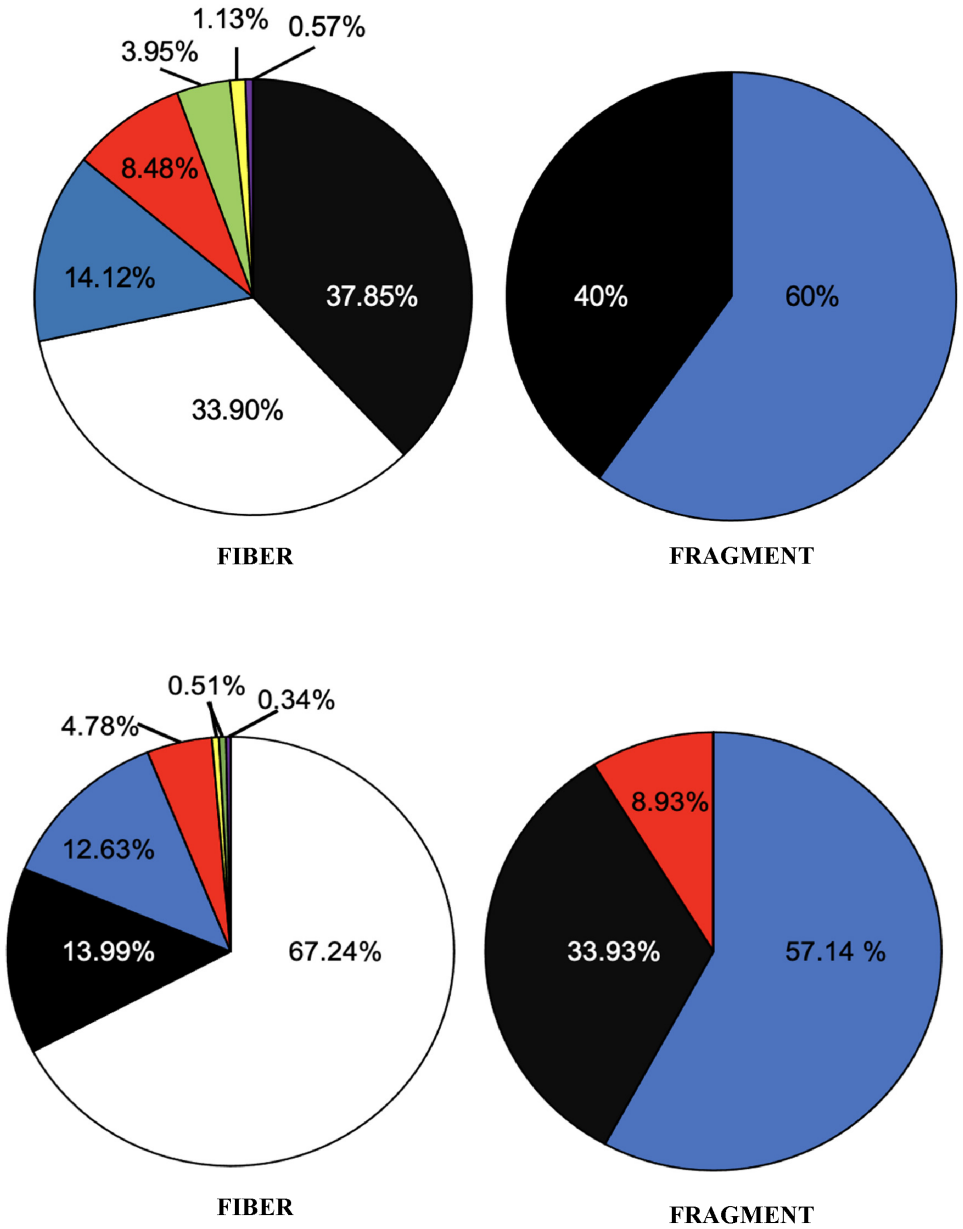
The total percentage of microplastic fibers, and microplastic fragments in all (a) water samples, and (b) *P. leniusculus* samples categorized by color. Twelve colorless microplastic films were also recorded in *P. leniusculus* samples.

Microplastics were recorded in *P. leniusculus* and water samples from every site included in this study. In total, 41 *P. leniusculus* (CL 24.4 mm – 54.0 mm) were caught across the eight sites: BGB (*n* = 16), BDN (*n* = 2), KEX (*n* = 3), KTW (*n* = 4), AUT (*n* = 3), CPT (*n* = 7), TWN (*n* = 4), EBN (*n* = 2). Microplastic fibers, films (Figure 2), and fragments were identified in *P. leniusculus* across all samples. Microplastic fibers and fragments were recorded in water samples. Across all samples, the total number of suspected microplastics identified was 841 (654 in crayfish; 187 in water samples). Fibers were most abundant; a total of 763 fibers from seven color categories were visually identified: white, black, blue, red, green, yellow, and purple. White fibers were most abundant in *P. leniusculus* samples, while black fibers were most abundant in water samples (Figure 3). In addition, 12 pieces of transparent film and 66 fragments from three color categories were visually identified: black, blue, and red. The randomly selected subsample represented 57 suspected microplastics from *P. leniusculus* samples and 22 particles from water samples. Of the 79 suspected microplastics analyzed through μFT‐IR, 54 particles (68.35%) were confirmed as plastic polymers with match similarity scores ≥70%, and 11 particles (13.92%) were confirmed as naturally sourced. The remaining 14 particles had match similarity scores that were deemed unreliable (<70%). In both crayfish and water samples, the most prevalent polymer types identified by μFT‐IR were polyester, epoxy resin, and polyethylene. Polyester was found in crayfish at five sites and in water at three sites. Particles identified as epoxy resin were found in crayfish at four sites and in water at three sites. Polyethylene was found in crayfish at four sites and in water at one site. Polyacrylonitrile was found in crayfish at three sites and in water at two sites. Cellophane was found in crayfish at three sites and in water at two sites (Appendix 1: Tables A1 and A2). Polyester was the most abundant particle representing 17.02% and 16.67% of microplastics in crayfish and water samples. Epoxy resin was the second most abundant particle representing 14.89% and 16.67% of microplastics in crayfish and water samples. Plastic polymers, compared with natural fibers, were more common in the vicinity of highly urbanized areas.

### 3.2 Microplastic contamination

**FIGURE 4:**
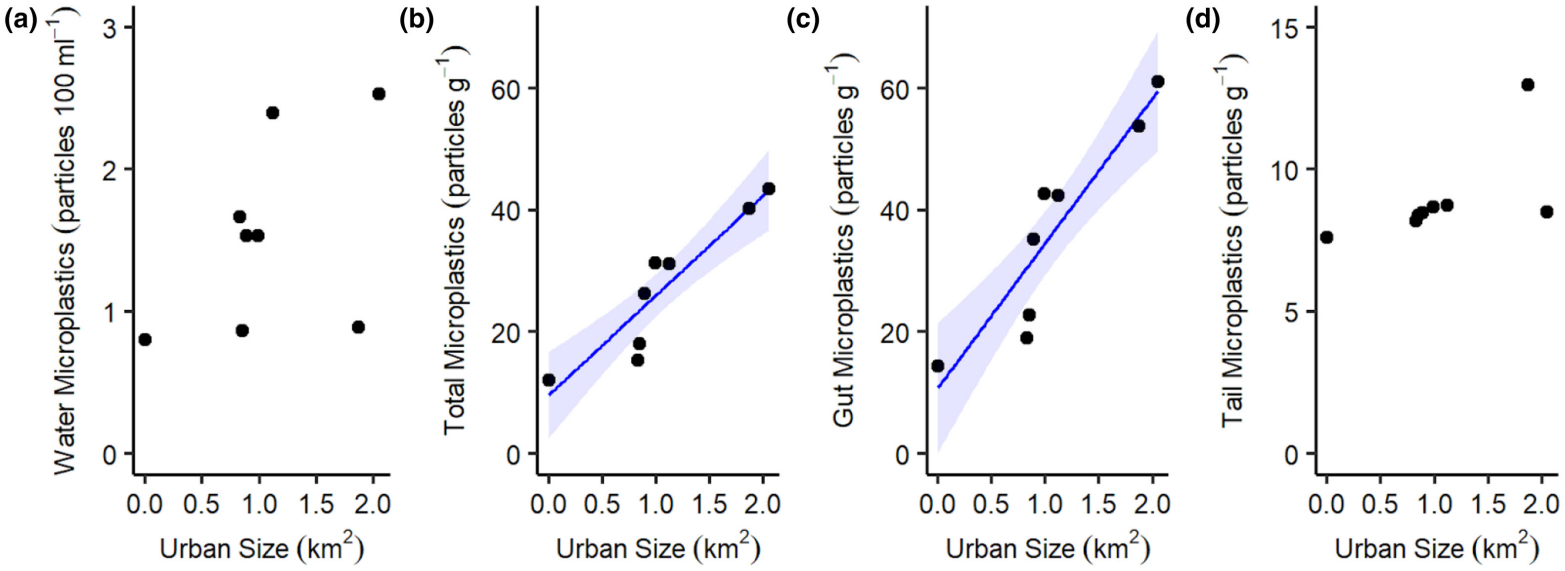
Scatter plots of microplastic concentration in water (a), total (b), gut (c), and tail (d) samples against urban area size in signal crayfish. Blue lines represent regressions where there is strong evidence (significant at *p* = .05) for urban area size to predict microplastic concentration, ribbons reflect confidence intervals (2 standard error).

Microplastics were found in all water samples collected at all sites, with a mean of 1.5 ± 0.7 microplastic pieces 100 mL^−1^ recorded. The highest concentration of microplastics was recorded at Eastburn and Captain Beck (2.8 pieces 100 mL^−1^) and the lowest was recorded at Bookill Gill Beck (0.6 pieces 100 mL^−1^). Generalized linear regression showed little to no evidence for a relationship between the concentration of microplastics in the water and urban area size (*F* = 1.649, *p* = .247; Figure 4).

Microplastics were seen in all *P. leniusculus* samples, in 100% of total gut samples, and 93% of tail samples, with a mean count of 16.1 microplastic particles per crayfish. Concentrations of microplastics in samples collected from individual signal crayfish ranged from 3.6 pieces g^−1^ in a crayfish from Bookill Gill Beck to 45.4 pieces g^−1^ from Eastburn Beck with a mean of 23.0 ± 11.4 pieces g^−1^ of crayfish. Generalized linear regression showed strong evidence (significant at *p* = .05) was available for a positive relationship between the total concentration of microplastics in *P. leniusculus* samples and urban area size (*F* = 32.478, *p* = .001; Figure 4).

Microplastic concentrations were highest in crayfish guts (mean = 30.2 ± 16.5 pieces g^−1^) and varied greatly between individual crayfish with 5.4 pieces g^−1^ in a crayfish from Bookill Gill Beck and 64.3 pieces g^−1^ in a crayfish from Eastburn Beck. Strong evidence (significant at *p* = .05) of a relationship between microplastic concentration within *P. leniusculus* guts and urban area (*F* = 30.451, *p* = .001) was also evident.

Microplastic concentrations in individual crayfish tails (mean = 8.6 ± 3.2 pieces g^−1^) were lower than in total or gut samples. Two crayfish tails at Bookill Gill Beck contained no microplastics while the highest concentration was recorded in a crayfish at Town Beck (13.9 pieces g^−1^ tail tissue). Little evidence (not significant at *p* = .05) was available for a linear relationship between *P. leniusculus* tail microplastic concentration and urban area (*F* = 4.531, *p* = .087).

**FIGURE 5:**
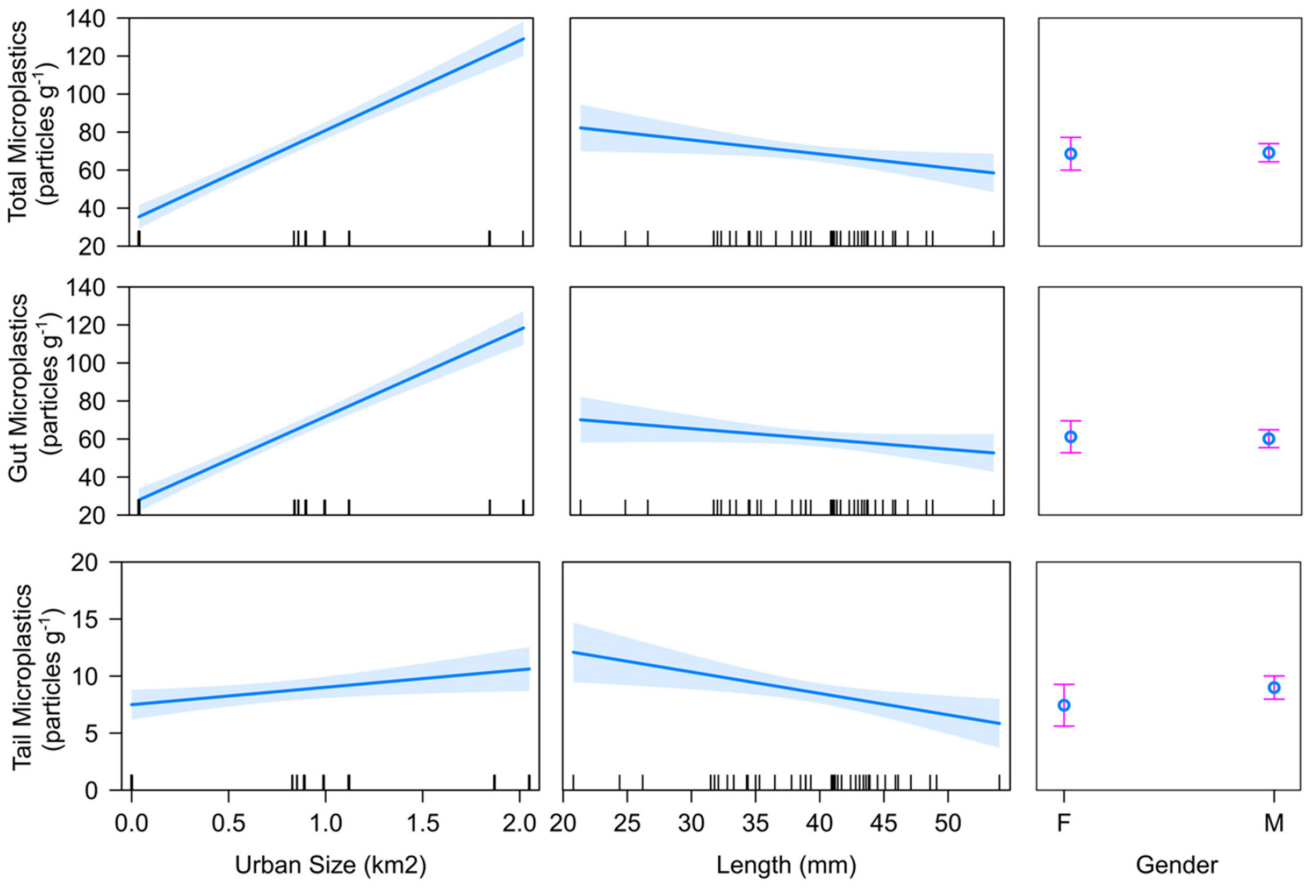
Effects plots for multiple regression models of signal crayfish microplastic concentrations against predictor variables. Top row = total microplastic concentration, middle row = gut microplastic concentration, and bottom row = tail microplastic concentrations.

In multiple generalized linear regression models, evidence was available for the effects of upstream catchment urbanization and *P. leniusculus* length of individuals on microplastic concentration. Strong evidence for a highly significant positive effect of urban area size on total crayfish microplastic concentration was found (*F* = 232.832, *p* < .001; Figure 5), as well as significant evidence of a negative effect of crayfish length (*F* = 5.548, *p* = .024). No evidence of an effect of crayfish gender was found (*F* = 0.013, *p* = .910). Strong evidence (significant at *p* = .05) of a positive effect of urban area size on gut microplastic concentration was found (*F* = 227.636, *p* < .001), but there was no evidence for an effect of crayfish length (*F* = 3.473, *p* = .070) and no evidence of an effect of crayfish gender (*F* = 0.042, *p* = .0.839). For tail microplastic concentrations a positive effect (significant at *p* = .05) of urban area size was evident (*F* = 6.956, *p* = .012), as well as a negative effect (significant at *p* = .05) of crayfish length (*F* = 6.495, *p* = .015). Again, no evidence of an effect of crayfish gender was found (*F* = 2.189, *p* = .147).

## 4 DISCUSSION

### 4.1 Microplastic occurrence and urbanization

To our knowledge, this research presents the first published evidence of microplastics in invasive *P. leniusculus* populations in Europe. It also reveals a ubiquitous presence of microplastics in headwater stream sites, including in catchments with almost no urbanization. Microplastics can be transported in a variety of ways and it is possible that atmospheric transport, degradation of litter (in situ or elsewhere followed by transport) and even very small semi‐urban areas can produce enough microplastics to be identified in adjacent ecosystems (Petersen & Hubbart, 2021). Our findings provide further empirical evidence for the ubiquity of microplastics and that both urban and rural land uses can be associated with their presence.

The concentration of microplastics in *P. leniusculus* was positively related to urban area size. Although sample sizes were small at some sites, within‐site standard deviations were small compared with means and the urban area size gradient. Further, the observed trend is in agreement with previous studies, which highlight a higher abundance of microplastic in freshwater species from urbanized locations (Parker et al., 2021; Peters & Bratton, 2016; Simmerman & Coleman Wasik, 2020), suggesting the observed trend reflects the underlying patterns reported across the literature. Stronger relationships between urban area and both total and crayfish gut microplastic samples were observed than for crayfish tail samples. Concentrations in crayfish tail tissue were low compared with those in total and gut samples and future work is required to determine whether a trend is present here. This may require larger sample sizes and a more extreme urbanization gradient than we were able to include here. In our study, the most urbanized sites were associated with small towns on the periphery of major urban conurbations. Maximum recorded crayfish microplastic burdens would likely be higher if more urbanized sites were selected to represent the upper extreme of the urbanization gradient.

Contrary to previous research (Hurley et al., 2018; Lebreton et al., 2017; McCormick et al., 2016), a relationship was not observed between microplastic abundance in water samples and urban areas. It is noted that water samples were collected in each catchment once on the day of crayfish sampling only. As a consequence, water samples were taken on different days under varying weather and flow conditions and this may be expected to introduce variability unrelated to urban area size (Hurley et al., 2018). Triplicate 0.5 liter water samples were filtered at each site and it is possible that if this volume was increased a stronger relationship may have been observed with urban area size. However, standard deviations in water sample microplastic concentrations were small compared with sample means at each site suggesting sufficient water was collected to reflect the point population means. The microplastic loading in water may follow anthropogenic temporal patterns for instance varying with inputs from wastewater treatment works and combined sewage overflows during rainfall events (Di Nunno et al., 2021; McCormick et al., 2016). Under such patterns, point sampling rather than repeated sampling risks under‐ or over‐reporting microplastic abundance as microplastic load in water can be expected to vary over very short time frames (< hours). Precedence therefore exists to use macroinvertebrates as bioindicators rather than waters to monitor pollutants such as nutrients (Ashton et al., 2014; Wright et al., 2000), sediment load (Extence et al., 2013), and flow conditions (Extence et al., 1999). It follows, therefore, that microplastic abundance in *P. leniusculus* digestive tracts may better reflect average microplastic loading within the ecosystem as material passes through the gut over a much longer period (e.g., being retained in the foregut alone for up to 9 h, Loya‐Javellana et al., 1995). As such, invasive crayfish may act as excellent indicator taxa for monitoring microplastic abundance in aquatic environments that demonstrate high variability in water microplastic abundance, although biosecurity and permitting should be considered. This idea, however, requires targeted research and in particular further assessment of other potential indicators such as sediment, primary producers, detritus, and other organisms with suitable feeding ecology.

Microplastic abundance reported here greatly exceeds those in previous research on crayfish. To the authors’ knowledge, only two previous studies have reported microplastic abundance within crayfish (Lv et al., 2019; Zhang, Fraser, et al., 2021) reporting mean abundances of 2.5 ± 0.6 and 0.92 ± 0.19 microplastic particles per crayfish compared with 16.1 ± 6.9 microplastic particles per crayfish in this study. This may be the result of higher environmental loads in the present study location. Lv et al. (2019) and Zhang, Fraser, et al. (2021) investigated microplastic pollution in isolated rice paddies and controlled freshwater aquaculture systems, respectively. Further research is required to elucidate how crayfish accumulate microplastics within different ecosystems, and the role of life histories and environmental conditions in this process (Alcorlo et al., 2004; Harvey et al., 2011; Souty‐Grosset et al., 2016).

Fibers were the most commonly identified microplastic morphology in our study, representing more than 90% of the total microplastic particles observed. Fibers are often the most commonly identified microplastic shape in many freshwater studies (Eerkes‐Medrano & Thompson, 2018; Tanentzap et al., 2021). For example, fibers in invertebrates were identified as the most common microplastic type found in some caddisflies (Gallitelli et al., 2021), mayflies (Akindele et al., 2020; Windsor et al., 2019), worms (Hurley et al., 2017) and freshwater shrimp (Nan et al., 2020). Our research extends this group to include *P. leniusculus*.

### 4.2 Microplastic and crayfish

In addition to urban areas, there was moderate evidence for a negative relationship between individual carapace length and microplastic abundance in crayfish. This finding is counterintuitive given a wealth of literature on the trophic transfer of microplastics through food webs (Athey et al., 2020; Costa et al., 2020; D'Souza et al., 2020) and crayfish occupying multiple trophic levels including engaging in cannibalism (Bondar et al., 2005; Rummel et al., 2017). Another explanation for this relationship may be driven by diet. Ontogenetic diet shifts in crayfish are observed with smaller crayfish more heavily relying on invertebrates, while larger crayfish consume greater amounts of detritus and plant material (Scalici & Gibertini, 2007). Evidence within the freshwater literature of increased microplastic concentrations with increased trophic levels supports this hypothesis (Mateos‐Cárdenas et al., 2022). However, recent evidence suggests diet changes as a function of seasonality rather than size (Ercoli et al., 2021). Therefore, future research is required to provide a comprehensive assessment of the inter‐ and intraspecific drivers of microplastic contamination in crayfish.

Microplastic contamination within the gastrointestinal tract of target species is almost ubiquitously reported across the literature (Gouin, 2020). Following ingestion, microplastics can pass through the digestive tract and be excreted or can translocate across the gut lining and persist in tissues (Browne et al., 2008; Carr et al., 2012; Messinetti et al., 2019). Translocation of microplastics into other organs and tissues, however, is much rarer and less consistently reported. To the authors' knowledge, microplastics found in *P. leniusculus* tail samples from this study provide the first evidence of such translocation into the body of crayfish. Evidence of translocation of microplastics into tissues has been provided for other aquatic taxa, such as livers in fish (Ding et al., 2018; Song et al., 2022) and muscle in tiger prawns *Penaeus semisulcatus* (Abbasi et al., 2018). However, some studies also report no evidence of translocation in sampled tissues, such as in the muscle tissue of commercial crab species (Zhang, Sun, et al., 2021) and muscle and liver tissues of commercial fish species (Su et al., 2019).

Translocation of microplastics is a prerequisite process for bioaccumulation and biomagnification to occur. Evidence of translocation of microplastics within wild‐caught crayfish provided within this study therefore provides crucial support for the inclusion of crayfish in future work exploring impacts of bioaccumulation and biomagnification processes, such as ecotoxicology (Anbumani & Kakkar, 2018; Mallik et al., 2021) and impaired physiological performance (Mkuye et al., 2022; Welden & Cowie, 2016). In our study, microplastics were observed within tail muscle tissue; however, no organs outside of the gastrointestinal tract were sampled. As such, it is clear that confirmation of whether microplastics can translocate into additional tissues within crayfish warrants further research. Furthermore, the exact physiological mechanism of translocation is not fully understood for larger microplastic particles (>200 μm) and requires further study, especially in larger organisms such as fish and crayfish (McIlwraith et al., 2021).

The size and shape of microplastic particles influence translocation, with small fibers translocating more readily (Browne et al., 2008). Furthermore, polymer type influences the toxicological effects of microplastic ingestion and translocation (Kögel et al., 2020; Rochman et al., 2019; Sheng et al., 2021). Chemical analysis of individual plastic particles identified polyester as the most common polymer type in *P. leniusculus* and water samples within this study, at 17.02% and 16.67%, respectively. Polyester, which accounts for more than half of the synthetic textile fibers produced globally, has been shown to cause cellular damage in mammal species, and decreased reproduction in soil invertebrates (Browne et al., 2008; Selonen et al., 2020). The translocation of polyester fibers may cause similar negative effects in crayfish; however, further research on the fate of microplastics and the contaminant loading of translocated polymers is required.

## 5 CONCLUSIONS

In conclusion, our study demonstrates microplastic contamination in crayfish for the first time in Europe. Further, it demonstrates a positive trend between microplastic concentration in crayfish and urban area size, extending a trend reported for a range of other species. Our results indicate much higher microplastic burdens in *P. leniusculus* within lotic systems than reported elsewhere for other crayfish species in aquaculture and lentic systems; however, the drivers of this remain unclear. An empirical study of in situ microplastic contamination, accumulation, and trophic transport in freshwaters is therefore essential but has been limited to date. To this end, our study provides novel in situ evidence of microplastic contamination and translocation in invasive crayfish in Europe.

## AUTHOR CONTRIBUTIONS

**Abigail Rose Dent:** Conceptualization (lead); data curation (lead); formal analysis (lead); funding acquisition (lead); investigation (lead); methodology (lead); project administration (lead); resources (lead); software (lead); writing – original draft (lead); writing – review and editing (lead). **Daniel Chadwick:** Formal analysis (equal); investigation (equal); methodology (equal); writing – original draft (equal); writing – review and editing (equal). **Lawrence Eagle:** Formal analysis (equal); investigation (equal); methodology (equal); writing – original draft (equal); writing – review and editing (equal). **Alex Gould:** Writing – original draft (equal); writing – review and editing (equal). **Neil Rose:** Supervision (equal); writing – review and editing (equal). **Carl D. Sayer:** Writing – review and editing (equal). **Matthew Harwood:** Investigation (equal); writing – review and editing (equal).

## Data Availability

The data that support the findings of this study are openly available in Dryad at [https://doi.org/10.5061/dryad.pk0p2ngt0].

## APPENDIX 1

**TABLE A1:** Compound names of fibers and fragments (with match similarity scores ≥70%) identified in *P. leniusculus* samples using FT‐IR. Natural polymers are in bold.

Study site	Compound name	*n*
BGB	Epoxy resin	1
	Polyester	1
	**Cellulose**	1
	Polypropylene	1
BDN	Cellophane	2
	**Cellulose + Lignin**	2
	Phenoxy resin	1
KEX	Polyvinyl alcohol	2
	Polyethylene	2
	**Wood**	1
KTW	Polyacrylonitrile	1
	Polyester	1
	Epoxy resin	1
	**Cellulose + Lignin**	1
AUT	Polyvinyl alcohol	2
	Cellophane	2
	Bakelite	1
	Polyacrylonitrile	1
	**Protein α‐helix**	1
CPT	Polyethylene	2
	Polyester	2
	Epoxy resin	2
	**Pinewood**	1
	Polyacrylonitrile	1
TWN	Epoxy resin	3
	Polyester	2
	Bakelite	1
	**Chipboard**	1
	Polyethylene	1
EBN	Polyethylene	2
	Polyester	2
	Cellophane	1
	Phenoxy resin	1

**TABLE A2:** Compound name of fibers and fragments (with match similarity scores ≥70%) identified in water samples using FT‐IR. Natural polymers are in bold.

Study site	Compound name	*n*
BGB	**Cellulose**	1
	Epoxy resin	1
	Polypropylene	1
BDN	Bakelite	1
	**Cellulose + Lignin**	1
KEX	Phenoxy resin	1
	Polyacrylonitrile	1
KTW	Polyacrylonitrile	1
	Polyester	1
AUT	Cellophane	1
	Epoxy resin	1
CPT	Cellophane	1
	Polyvinyl alcohol	1
TWN	**Cellulose**	1
	Polyester	1
EBN	Epoxy resin	1
	Polyester	1
	Polyethylene	1
